# Diversity and distribution of *Actinobacteria* associated with reef coral *Porites lutea*

**DOI:** 10.3389/fmicb.2015.01094

**Published:** 2015-10-21

**Authors:** Weiqi Kuang, Jie Li, Si Zhang, Lijuan Long

**Affiliations:** ^1^CAS Key Laboratory of Tropical Marine Bio-Resources and Ecology, RNAM Center for Marine Microbiology, South China Sea Institute of Oceanology, Chinese Academy of SciencesGuangzhou, China; ^2^College of Earth Science, University of Chinese Academy of SciencesBeijing, China

**Keywords:** actinobacteria, *Porites lutea*, diversity, temporal and spatial distribution, 16S rRNA gene

## Abstract

*Actinobacteria* is a ubiquitous major group in coral holobiont. The diversity and spatial and temporal distribution of actinobacteria have been rarely documented. In this study, diversity of actinobacteria associated with mucus, tissue and skeleton of *Porites lutea* and in the surrounding seawater were examined every 3 months for 1 year on Luhuitou fringing reef. The population structures of the *P. lutea*-associated actinobacteria were analyzed using phylogenetic analysis of 16S rRNA gene clone libraries, which demonstrated highly diverse actinobacteria profiles in *P. lutea*. A total of 25 described families and 10 unnamed families were determined in the populations, and 12 genera were firstly detected in corals. The *Actinobacteria* diversity was significantly different between the *P. lutea* and the surrounding seawater. Only 10 OTUs were shared by the seawater and coral samples. Redundancy and hierarchical cluster analyses were performed to analyze the correlation between the variations of actinobacteria population within the divergent compartments of *P. lutea*, seasonal changes, and environmental factors. The actinobacteria communities in the same coral compartment tended to cluster together. Even so, an extremely small fraction of OTUs was common in all three *P. lutea* compartments. Analysis of the relationship between actinobacteria assemblages and the environmental parameters showed that several genera were closely related to specific environmental factors. This study highlights that coral-associated actinobacteria populations are highly diverse, and spatially structured within *P. lutea*, and they are distinct from which in the ambient seawater.

## Introduction

Coral reef ecosystem is one of the most important tropical marine ecosystems, mainly distributed in the Indo-West Pacific, Eastern Pacific, Western Atlantic, and the Eastern Atlantic (Moberg and Folke, 1999). Corals provide habitats for numerous bacteria in their mucus layer, tissue, and calcium carbonate skeleton, as well as the dinoflagellates, fungi, archaea, and viruses (Rosenberg et al., 2007). Coral-associated bacteria not only take part in carbon, nitrogen, and sulfur biogeochemical cycles and provide necessary nutrient for coral, but also keep corals from being infected by pathogens (Rosenberg et al., 2007; Raina et al., 2009; Bourne and Webster, 2013).

Highly diverse and heterogeneous bacterial communities have been revealed in different coral species at various locations (Rohwer et al., 2002; Li et al., 2013). *Actinobacteria* is generally accepted as a ubiquitous major group in corals (Bourne and Munn, 2005; Carlos et al., 2013; Li et al., 2013, 2014a). Yang et al. (2013) detected 19 *Actinobacteria* genera in soft coral *Alcyonium gracllimum* and stony coral *Tubastraea coccinea* in the East China Sea through analysis of 16S rRNA gene clone libraries. Some actinobacterial genera were previously detected in corals by using the culture-dependent method (Lampert et al., 2006; Nithyanand and Pandian, 2009; Nithyanand et al., 2011b; Zhang et al., 2013; Li et al., 2014b). Among these culturable actinobacteria, *Streptomyces, Verrucosispora, Rhodococcus, Micromonospora, Nocardia, Jiangella, Nocardiopsis, Pseudonocardia*, and *Salinispora* showed antibacterial activities, which were considered to contribute to coral health (Ritchie, 2006; Nithyanand et al., 2011a; Krediet et al., 2013; Zhang et al., 2013; Li et al., 2014b).

Environmental conditions, coral species, colony physiology, and seasonal variation are considerable influencing factors on the coral-associated bacterial community (Hong et al., 2009). Moreover, due to various microhabitats provided by corals' biological structures, the spatial heterogeneity has been proved in bacterial communities associated with a single coral colony (Rohwer et al., 2002; Sweet et al., 2011; Li et al., 2014a). As a major coral-associated bacterial group, how actinobacteria is spatially and temporally organized in corals, and what is the connection between the actinobacteria communities in corals and in seawater remains poorly understood. Comprehensive investigation of the distribution of this ubiquitous group at spatial and temporal scales will help understanding the variation of coral associated bacteria and the potential function of actinobacteria, and will contribute a lot to bioprospect the actinobacteria resources for utilization as novel sources for bioactive natural products.

Coral reefs are widely distributed in the South China Sea (Liu et al., 2009; Wang et al., 2014). *Porites lutea* is the dominant, typical coral species in the Luhuitou fringing reef, which is located in the south end of the Hainan province (Zhao et al., 2008). In this study, the diversity and distribution of actinobacteria were investigated in coral *P. lutea* and in the surrounding seawater every 3 months for 1 year using culture-independent method for the first time. We aimed to reveal the coral-associated actinobacteria community structures in three divergent coral compartments in different months, compare the actinobacterial communities in the coral and in the surrounding seawater, and research the actinobacteria community variation responds to the environmental factors.

## Materials and methods

### Sample collection

The coral and surrounding sea water samples were collected in four different months (February, May, August, and November) in 2012 from the Luhuitou fringing reef (109°28′E, 18°13′N). Coral fragments (approximately 10 × 10 cm) were collected from the side of three healthy *P*. *lutea* colonies at the depth of 3–5 m each time using punch and hammer. Coral mucus, tissues and skeleton were separated and stored according to the method described previously (Li et al., 2014a). One liter of seawater adjacent to the coral colonies was collected, and filtered through 0.22 μm-pore-size filter membrane (Millipore). The filter membranes were stored at −80°C until DNA extraction. As the samples were collected at the same time, environmental parameters including water temperature, salinity, dissolved oxygen, pH value, ultraviolet radiation intensity, and rainfall were cited from the published data (Li et al., 2014a).

### DNA extraction and PCR amplification

The coral tissue and skeleton samples were homogenized thoroughly in liquid nitrogen with sterile mortar and pestle before added to the PowerBead Tubes. The filter membranes with adsorbed microbial cells were cut into pieces, and then added to the PowerBead Tubes. Total DNA was extracted using the PowerSoil DNA Isolation Kit (MoBio, Solana Beach, CA, USA) according to the manufacturer's instruction.

16S rRNA genes were nest PCR amplified, the first PCR reactions using the combination of universal bacterial primers 27F (5′-AGAGTTTGATCMTGGCTCAG-3′) and 1492R (5′-TACGGYTACCTTGTTACGACTT-3′). PCR amplifications were performed in a Mastercycler pro (Eppendorf, Hamburg, Germany) in a final volume of 50 μL, containing 2 μL (10 μM) each primer, 1 μL (10–20 ng) template DNA and 25 μL premix *Ex Taq* mixture (Takara, Dalian). The PCR conditions were as follows: 94°C for 5 min; 30 cycles of 94°C for 30 s, 54°C for 30 s, 72°C for 90 s; followed by 72°C for 10 min. In the second PCR reactions, the actinobacteria-specific primer pairs, S-C-Ac-0325-a-S-20 (5′-CGCGCCTATCAGCTTGTTG-3′) and S-C-Act-0878-a-A-19 (5′-CCGTATCCCCAGGCGGGG-3′), were used to amplify the V3-V5 regions (about 640 bp) of the actinobacteria 16S rRNA gene (Stach et al., 2003). In the PCR reactions, 5 μL of 1: 10 dilution of the first round PCR product was used as DNA template, the PCR mixture (50 μL) contain 2 μL (10 μM) each primer, 25 μL premix *Ex Taq* mixture, the PCR conditions were as follows: 95°C for 5 min; 30 cycles of 95°C for 45 s, 68°C for 45 s, 72°C for 60 s; followed by 72°C for 10 min. Each genomic DNA sample was amplified in triplicate PCR reactions. Amplicons from the same sample were pooled and purified using the E.Z.N.A.® Gel Extraction Kit (Omega Bio-Tek, China).

### Gene library construction and sequencing

Sixteen clone libraries of actinobacterial 16S rRNA genes were constructed using the pMD18-T Vector Cloning Kit and *E. coli* DH5α competent cells (Takara, Dalian) following the manufacturer′s instructions. The positive clones from each library inoculated on MacConkey agar with ampicillin (100 μg/ml) were randomly picked and sequenced using M13F (−47) primer on ABI 3730xl capillary sequencers (Applied Biosystems, USA).

### Libraries analysis

The vector sequences were screened by the VecScreen tool provided in NCBI (http://www.ncbi.nlm.nih.gov/tools/vecscreen/). Chimeras were checked by running chimera.uchime packaged in Mothur (Schloss et al., 2009), and potential chimeras were removed. All valid sequences were deposited in GenBank (accession numbers were shown in Data S1). All qualified sequences were identified by using the classify.seqs command in Mothur with Silva reference alignment database (http://www.mothur.org/wiki/Silva_reference_files, Release 119) at a confidence level of 80%. The sequences, which do not belong to *Actinobacteria*, were removed from further analysis. Sequences were clustered into operational taxonomic units (OTUs) with a 97% threshold using the cluster command in Mothur. The relationships among actinobacterial communities associated with different coral compartments and in the ambient seawater in different months were analyzed by hierarchical cluster analysis. Based on Bray-Curtis similarity estimated from the OTU matrix, clustering was generated by using the complete linkage method with the PRIMER 5 software (Clarke, 1993). The shared OTUs were determined by using the online tool venny (Oliveros, 2007–2015, http://bioinfogp.cnb.csic.es/tools/venny/index.html).

The correlations between *Actinobacteria* assemblages of coral samples and the environmental factors were analyzed by using the software package CANOCO 4.5.1 (ter Braak and Šmilauer, 2002). Redundancy analysis (RDA) was carried out to determine the relationship between the actinobacteria community and the environmental factors including temperature, salinity, dissolved oxygen, pH value, rainfall, and UV radiation and in combination with two nominal variables including the coral divergent compartments and the different sampling months. The significance of the relation between the explanatory variables and the actinobacterial community compositions was tested using Monte Carlo permutation tests (9999 unrestricted permutations, *P* < 0.05).

## Results

### Coral-associated actinobacteria diversity

**Figure 1:**
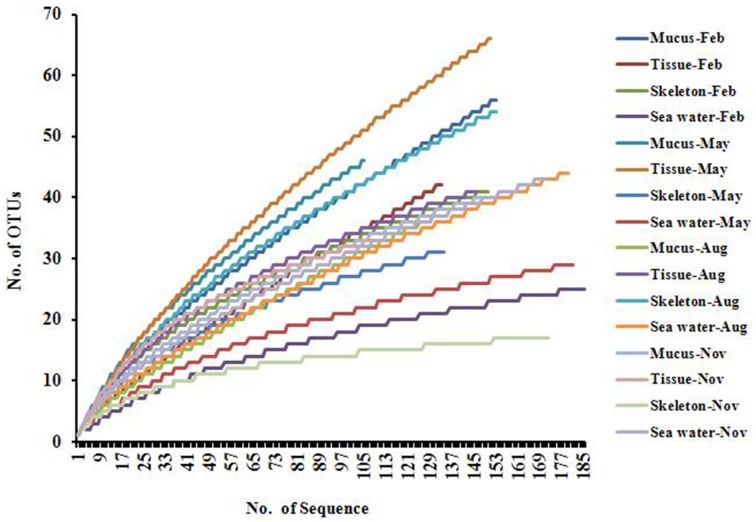
**Rarefaction curves of ***Actinobacteria*** 16S rRNA gene sequences**.

A total of 2403 sequences were obtained from sixteen 16S rRNA gene clone libraries, resulting in 395 OTUs (stringency at 97%). The rarefaction analysis result showed that most of the curves did not flatten to asymptote, but climbed less steeply (Figure 1). The coverages ranged from 0.69 to 0.97 in 16 libraries, and the average coverage was 0.83 (Table 1). The highest number of OTUs was found in the tissue collected in May, while the lowest OTUs was found in the skeleton collected in November (Table 1). The Shannon indices in mucus collected in different months ranged from 2.32 to 3.44, from 2.45 to 3.55 in tissues, from 1.82 to 3.35 in skeleton, and from 1.53 to 2.82 in sea water (Table 1), and the diversity in the actinobacterial community associated with *P. lutea* was higher than which in the surrounding sea water (*P* = 0.045).

**Table 1:** **Number of sequences and OTUs (97%) and diversity estimates of the ***Actinobacteria*** libraries in ***P***. ***lutea*** and in the ambient seawater**.

**Index**	**A1**	**A2**	**A3**	**A4**	**B1**	**B2**	**B3**	**B4**	**C1**	**C2**	**C3**	**C4**	**D1**	**D2**	**D3**	**D4**
No. of Seq.	153	133	150	185	105	151	134	181	132	146	153	179	149	109	172	171
OTUs	56	42	41	25	46	66	31	29	37	41	54	44	40	33	17	43
Chao	343.00	147.60	69.88	34.43	108.14	201.13	44.00	55.25	63.86	64.75	124.13	106.14	55.83	48.17	19.50	66.00
ACE	600.00	756.54	131.72	56.36	194.33	388.96	61.80	63.38	535.51	93.22	182.95	108.04	68.08	46.83	21.10	114.38
Shannon	3.33	2.45	3.08	1.53	3.44	3.55	2.70	1.89	2.32	3.07	3.35	2.68	2.89	3.11	1.82	2.84
Coverage	0.73	0.75	0.85	0.94	0.71	0.69	0.90	0.92	0.79	0.86	0.78	0.83	0.87	0.87	0.97	0.86

*A1, mucus in February; A2, tissue in February; A3, skeleton in February; A4, seawater in February; B1, mucus in May; B2, tissue in May; B3, skeleton in May; B4, seawater in May; C1, mucus in August; C2, tissue in August; C3, skeleton in August; C4, seawater in August; D1, mucus in November; D2, tissue in November; D3, skeleton in November; D4, seawater in November*.

### Coral-associated actinobacterial community composition

At a confidence threshold of 80%, 2403 qualified reads were assigned to four classes, i.e., *Acidimicrobiia, Actinobacteria, Thermoleophilia*, and KIST-JJY010. Among them, *Acidimicrobiia* and *Actinobacteria* were ubiquitous and dominant in *P. lutea* and in the seawater samples. *Thermoleophilia* was not detected in corals collected in February, in the mucus and seawater in May, and in the mucus in August, while accounted for 0.5–48.8% in all other samples. KIST-JJY010 was detected only in the mucus in November (0.6%), and in the skeleton in August (2.6%).

**Figure 2:**
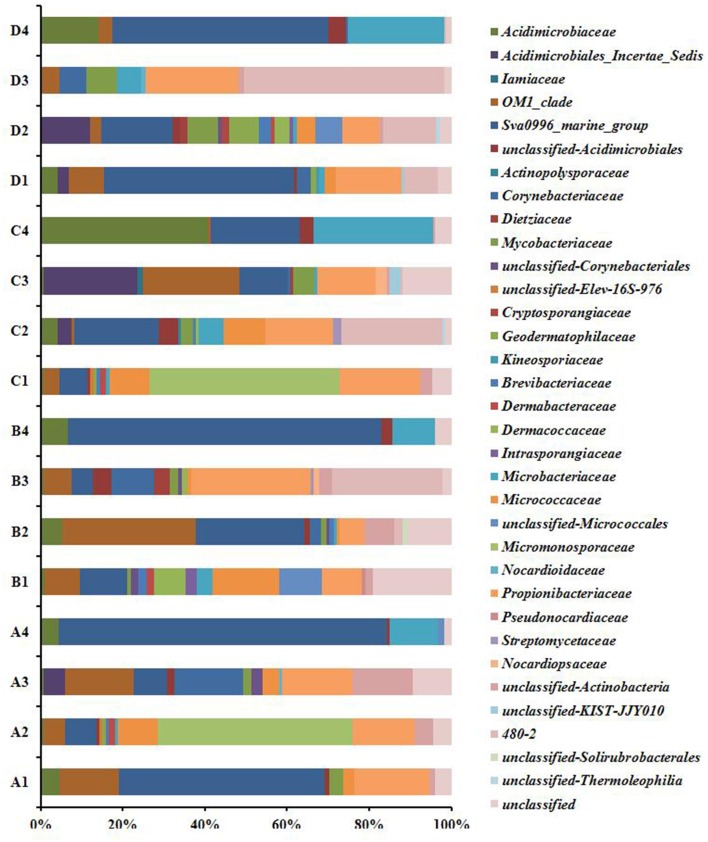
*****Actinobacteria*** composition profiles**. Taxonomic classification of actinobacteria sequences in to family identified by using the classify.seqs command in Mothur using Silva reference alignment database (http://www.mothur.org/wiki/Silva_reference_files, Release 119) with a confidence level of 80% were applied for classification. A1, mucus in February; A2, tissue in February; A3, skeleton in February; A4, seawater in February; B1, mucus in May; B2, tissue in May; B3, skeleton in May; B4, seawater in May; C1, mucus in August; C2, tissue in August; C3, skeleton in August; C4, seawater in August; D1, mucus in November; D2, tissue in November; D3, skeleton in November; D4, seawater in November.

Twenty-five described families and 10 unnamed families were detected in the 16 libraries (Figure 2). OM1_clade and *Propionibacteriaceae* (genera *Friedmanniella* and *Propionibacterium*) were ubiquitous, major groups in *P. lutea*. Meanwhile, OM1_clade was not detected in the seawater in February and May, and rare in the other two seawater libraries, and *Propionibacteriaceae* was absent in all the seawater libraries. *Micromonosporaceae* was the most abundant group in the tissue in February (47.4%) and in the mucus in August (46.2%), in which most of the reads were affiliated with an unclassified group. Nonetheless, *Micromonosporaceae* was absent in all other coral and seawater samples. Sva0996_marine_group was detected in all coral samples (5.2–50%) except in the skeleton collected in November, and which also was abundant in the ambient sea water (21.9–80%). *Micrococcaceae* was absent in the coral skeleton collected in August and in November, and in the sea water samples. Group 480-2 was abundant in the coral tissue in August (24.7%), as well as in the skeleton in May (26.9%) and in November (48.8%), but it was nearly absent in the surrounding seawater. In reverse, *Microbacteriaceae* and *Ilumatobacter* were major groups in sea water, while they were less abundant in *P. lutea*.

### Spatial and temporal distribution of *P. lutea*-associated actinobacteria

**Figure 3:**
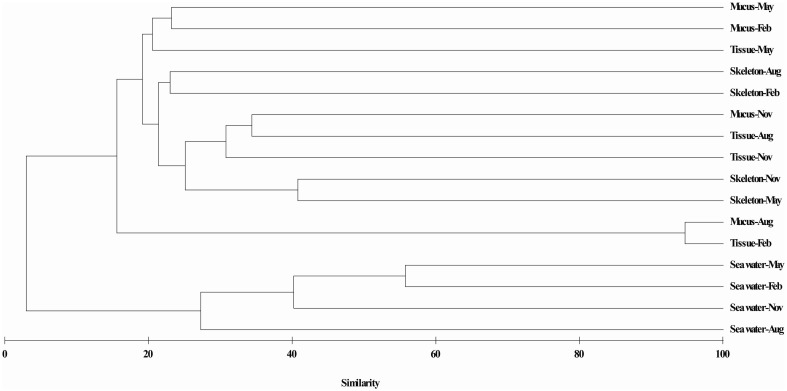
**Hierarchical cluster analysis of actinobacteria communities associated with ***P. lutea*****. Clustering was based on Bray-Curtis similarity estimated from the OTUs matrix by using the complete linkage method.

**Figure 4:**
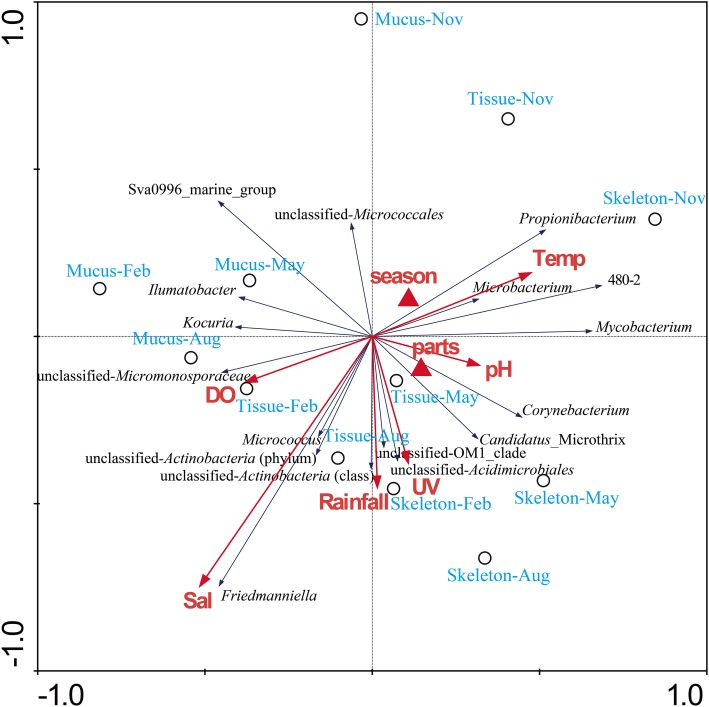
**RDA ordination triplot showing the relationship among the environmental variables, coral samples, and actinobacterial components**. Correlations between environmental variables and the first two RDA axes are represented by the lengths and angles of the arrows (environmental-factor vectors). Only abundant actinobacterial groups (>1%) were showed in the triplot. UV, ultraviolet radiation intensity; Temp, seawater temperature; DO, dissolved oxygen.

Results of hierarchical cluster analysis showed that the actinobacteria communities were significantly different between in the coral and in the surrounding seawater samples (*p* = 0.01, *R* = 0.993). The actinobacterial communities associated with the same coral compartments tended to cluster together (Figure 3). The season factor did not significantly influence the variation in the actinobacteria communities. The RDA results indicated that 38.9% of the total variance in the coral-associated actinobacterial composition was explained by the environmental, spatial and temporal factors (Figure 4). The first and second axes differentiated the actinobacteria assemblages in the distinct coral compartments (Figure 4, Table S1). This result was consistent with the hierarchical cluster analysis. None of the environment parameters analyzed in this study was determined as the significant influencing factor in the variation of the *P*. *lutea* associated actinobacteria communities. A triplot map illustrated the relationship between major actinobacterial groups, with abundance more than 1%, and the environmental parameters (Figure 4). *Friedmanniella* and *Micrococcus* were positively related with the salinity. *Microbacterium, Propionibacterium*, and group 480-2 were positively correlated with seawater temperature, but negatively correlated with dissolved oxygen.

To investigate the distribution of OTUs in the three divergent coral compartments (mucus, tissue, and skeleton) and in the surrounding seawater, a venn diagram was constructed. The results showed that only 5 OTUs were present in all of *P. lutea* mucus, tissue and skeleton, and in sea water, which were identified as Sva0996_marine_group, *Ilumatobacter, Corynebacterium*, OM1_clade and *Microbacterium* (Table 2, Figure S1A). Another 17 OTUs, which were identified as *Candidatus*_Microthrix, *Corynebacteriales, Friedmanniella, Micrococcus, Mycobacterium*, OM1_clade, *Propionibacterium*, Sva0996_marine_group, *Yonghaparkia* and 480-2 were common in mucus, tissue, and skeleton (Table 2, Figure S1A). Twelve OTUs distributed in *Propionibacterium, Friedmanniella*, OM1_clade, Sva0996_marine_group, *Kocuria, Mycobacterium, Corynebacteriales, Brevibacterium*, and *Brachybacterium* were present in coral libraries in all four different months (Table 3, Figure S1B). The most abundant OTU0003, which was classified as *Propionibacterium*, was present in all coral samples with a high abundance (128 out of total 1687 reads in the coral libraries, 7.6%). The secondary abundance OTU0004 affiliated with *Friedmanniella* was present in all libraries except in skeleton collected in November.

**Table 2:** **OTUs presented in all of the coral and seawater libraries, or presented in all three divergent compartments of ***P. lutea*****.

**OTUs**	**Observed in samples**	**Abundance**	**Phylogenetic affiliation**
OTU0001	Mucus, Tissue, Skeleton, Sea water	303	Sva0996_marine_group
OTU0007	Mucus, Tissue, Skeleton, Sea water	63	*Ilumatobacter*
OTU0011	Mucus, Tissue, Skeleton, Sea water	46	*Corynebacterium*
OTU0017	Mucus, Tissue, Skeleton, Sea water	33	OM1_clade
OTU0020	Mucus, Tissue, Skeleton, Sea water	24	*Microbacterium*
OTU0002	Mucus, Tissue, Skeleton	186	480-2
OTU0003	Mucus, Tissue, Skeleton	128	*Propionibacterium*
OTU0004	Mucus, Tissue, Skeleton	122	*Friedmanniella*
OTU0009	Mucus, Tissue, Skeleton	52	*Candidatus*_Microthrix
OTU0012	Mucus, Tissue, Skeleton	43	OM1_clade
OTU0013	Mucus, Tissue, Skeleton	40	OM1_clade
OTU0014	Mucus, Tissue, Skeleton	40	Sva0996_marine_group
OTU0023	Mucus, Tissue, Skeleton	21	*Micrococcus*
OTU0025	Mucus, Tissue, Skeleton	18	OM1_clade
OTU0027	Mucus, Tissue, Skeleton	18	*Mycobacterium*
OTU0028	Mucus, Tissue, Skeleton	17	*Corynebacteriales*
OTU0030	Mucus, Tissue, Skeleton	15	*Propionibacterium*
OTU0032	Mucus, Tissue, Skeleton	13	*Mycobacterium*
OTU0034	Mucus, Tissue, Skeleton	12	Sva0996_marine_group
OTU0035	Mucus, Tissue, Skeleton	12	Sva0996_marine_group
OTU0042	Mucus, Tissue, Skeleton	8	Sva0996_marine_group
OTU0056	Mucus, Tissue, Skeleton	5	*Yonghaparkia*

**Table 3:** **OTUs presented in ***P. lutea*** collected in four different months**.

**OTUs**	**Coral samples**	**Abundance**	**Phylogenetic affiliation**
OTU0003^a^	Feb, May, Aug, Nov	128	*Propionibacterium*
OTU0004	Feb, May, Aug, Nov	122	*Friedmanniella*
OTU0013	Feb, May, Aug, Nov	40	OM1_clade
OTU0014	Feb, May, Aug, Nov	40	Sva0996_marine_group
OTU0015	Feb, May, Aug, Nov	39	*Kocuria*
OTU0017	Feb, May, Aug, Nov	33	OM1_clade
OTU0022	Feb, May, Aug, Nov	21	Sva0996_marine_group
OTU0025	Feb, May, Aug, Nov	18	OM1_clade
OTU0027	Feb, May, Aug, Nov	18	*Mycobacterium*
OTU0028	Feb, May, Aug, Nov	17	*Corynebacteriales*
OTU0033	Feb, May, Aug, Nov	13	*Brevibacterium*
OTU0059	Feb, May, Aug, Nov	5	*Brachybacterium*

a *OTU0003 was present in all 12 libraries. The other OTUs listed in this table were present in either of the compartment mucus, tissue and skeleton of corals collected in four different months*.

## Discussion

### Highly diverse actinobacteria associated with *P. lutea*

In comparison with previously reported results (Lampert et al., 2006, 2008; Bruck et al., 2007; Kageyama et al., 2007; Santiago-Vázquez et al., 2007; Ben-Dov et al., 2009; Nithyanand and Pandian, 2009; Seemann et al., 2009; Shnit-Orland and Kushmaro, 2009; de Castro et al., 2010; Thomas et al., 2010; Nithyanand et al., 2011a,b; Cardenas et al., 2012; Chiu et al., 2012; Sun et al., 2012, 2014; Zhang et al., 2012, 2013; Yang et al., 2013; Chen et al., 2014; Li et al., 2014a,b; EIAhwany et al., 2015; Sarmiento-Vizcaíno et al., 2015), 12 genera including *Actinopolyspora, Blastococcus, Candidatus*_Aquiluna, *Demetria, Fodinicola, Friedmanniella, Geodermatophilus, Iamia, Modestobacter, Ornithinimicrobium, Tersicoccus*, and *Yonghaparkia* were firstly detected in corals in this study (Table 4). Furthermore, many unclassified groups were detected in *P. lutea*, including even the group at the class taxon level. These results suggested that highly diverse and abundant known actinobacteria were associated with *P. lutea* as well as unknown groups. It was also noticed that many actinobacterial groups were only detected by the culture-independent method (Table 4), and some of them were ubiquitous and abundant, such as *Friedmanniella, Ilumatobacter*, and OM1_clade. Their physiological properties and ecological significance are worthy of deep research. For this purpose, the development and innovation of the isolation and cultivation methods in order to obtain pure cultures from the coral holobiont is particularly important.

**Table 4:** **Summary of the *Actinobacteria* associated with corals**.

**Family**	**Genus**	**Source coral**	**Isolate/clone**	**References**
*Acidimicrobiaceae*	*Ilumatobacter*	*Porites lutea*	Clone	Chen et al., 2014
		*Porites lutea*	Clone	This study
***Iamiaceae***	***Iamia***	*Porites lutea*	Clone	This study
***Actinopolysporaceae***	***Actinopolyspora***	*Porites lutea*	Clone	This study
*Actinospicaceae*	*Actinospica*	Zoanthid *Palythoa australiae*	Clone	Sun et al., 2014
*Brevibacteriaceae*	*Brevibacterium*	*Acropora digitifera*	Isolate	Nithyanand and Pandian, 2009
		*Tubastraea coccinea*	Clone	Yang et al., 2013
		*Acropora millepora*	Isolate	Li et al., 2014b
		*Galaxea fascicularis*	Isolate	Li et al., 2014b
		*Porites lutea*	Isolate	Li et al., 2014b
		*Porites lutea*	Clone	This study
*Dermacoccaceae*	***Demetria***	*Porites lutea*	Clone	This study
	*Dermacoccus*	*Tubastraea coccinea*	Clone	Yang et al., 2013
	*Kytococcus*	*Fungia scutaria*	Isolate	Lampert et al., 2006
		*Porites lutea*	Clone	This study
*Dietziaceae*	*Dietzia*	*Leptogorgia minimata*	Isolate	Bruck et al., 2007
		*Scleronephthya* sp.	Isolate	Sun et al., 2012
		*Alcyonium gracllimum*	Clone	Yang et al., 2013
		*Tubastraea coccinea*	Clone	Yang et al., 2013
		Zoanthid *Palythoa australiae*	Clone	Sun et al., 2014
		*Porites lutea*	Clone	This study
***Geodermatophilaceae***	***Blastococcus***	*Porites lutea*	Clone	This study
	***Geodermatophilus***	*Porites lutea*	Clone	This study
	***Modestobacter***	*Porites lutea*	Clone	This study
*Intrasporangiaceae*	*Janibacter*	*Acropora gemmifera*	Isolate	Kageyama et al., 2007
		*Alcyoniu gracllimum*	Clone	Yang et al., 2013
		*Acropora gemmifera*	Isolate	Valliappan et al., 2014
		*Porites lutea*	Clone	This study
	***Ornithinimicrobium***	*Porites lutea*	Clone	This study
	*Serinicoccus*	*Tubastraea coccinea*	Clone	Yang et al., 2013
*Mycobacteriaceae*	*Mycobacterium*	*Sinularia* sp.	Isolate	Thomas et al., 2010
		*Scleronephthya* sp.	Isolate	Sun et al., 2012
		*Alcyoniu gracllimum*	Clone	Yang et al., 2013
		*Tubastraea coccinea*	Clone	Yang et al., 2013
		*Porites lutea*	Isolate	Li et al., 2014b
		*Porites lutea*	Clone	This study
*Nocardiaceae*	*Rhodococcus*	*Iciligorgia schrammi*	Isolate	Bruck et al., 2007
		*Scleronephthya* sp.	Isolate	Sun et al., 2012
		*Tubastraea coccinea*	Clone	Yang et al., 2013
*Nocardioidaceae*	*Nocardioides*	*Palythoa caribaeorum*	Isolate	Seemann et al., 2009
		*Scleronephthya* sp.	Isolate	Sun et al., 2012
		*Tubastraea coccinea*	Clone	Yang et al., 2013
		*Porites lutea*	Clone	This study
*Nocardiopsaceae*	*Nocardiopsis*	*Platygyra lamellina*	Clone	Lampert et al., 2008
		*Acropora millepora*	Isolate	Li et al., 2014b
		*Galaxea fascicularis*	Isolate	Li et al., 2014b
		*Porites lutea*	Isolate	Li et al., 2014b
		*Porites lutea*	Clone	This study
*Propionibacteriaceae*	***Friedmanniella***	*Porites lutea*	Clone	This study
	*Propionibacterium*	*Cirrhipiathes lutkeni*	Isolate	Santiago-Vázquez et al., 2007
		*Mussimilia hispida*	Isolate	de Castro et al., 2010
		*Acropora digitifera*	Isolate	Nithyanand et al., 2011b
		Zoanthid *Palythoa australiae*	Clone	Sun et al., 2014
		*Porites lutea*	Clone	This study
	*Tessaracoccus*	*Porites lutea*	Clone	Chen et al., 2014
*Pseudonocardiaceae*	*Pseudonocardia*	*Acropora millepora*	Isolate	Li et al., 2014b
		*Galaxea fascicularis*	Isolate	Li et al., 2014b
		Zoanthid *Palythoa australiae*	Clone	Sun et al., 2014
		*Porites lutea*	Clone	This study
	*Amycolatopsis*	*Galaxea fascicularis*	Isolate	Li et al., 2014b
		Zoanthid *Palythoa australiae*	Clone	Sun et al., 2014
	*Prauserella*	*Galaxea fascicularis*	Isolate	Li et al., 2014b
	*Saccharomonospora*	*Antipathes dichotoma*	Isolate	Seemann et al., 2009
*Streptomycetaceae*	*Streptomyces*	*Iciligorgia schrammi*	Isolate	Bruck et al., 2007
		*Acropora digitifera*	Isolate	Nithyanand et al., 2011b
		*Antipathes dichotoma*	Isolate	Zhang et al., 2012
		*Scleronephthya* sp.	Isolate	Sun et al., 2012
		*Alcyonium gracllimum*	Clone	Yang et al., 2013
		*Tubastraea coccinea*	Clone	Yang et al., 2013
		Zoanthid *Palythoa australiae*	Clone	Sun et al., 2014
		*Acropora millepora*	Isolate	Li et al., 2014b
		*Galaxea fascicularis*	Isolate	Li et al., 2014b
		*Porites lutea*	Isolate	Li et al., 2014b
		*Sarcophyton glaucum*	Isolate	EIAhwany et al., 2015
		*Porites lutea*	Clone	This study
*Cellulomonadaceae*	*Cellulomonas*	*Scleronephthya* sp.	Isolate	Sun et al., 2012
		*Alcyomum gracllimum*	Clone	Yang et al., 2013
		Zoanthid *Palythoa australiae*	Clone	Sun et al., 2014
*Dermatophilaceae*	*Dermatophilus*	*Fungia scutaria*	Isolate	Lampert et al., 2006
		*Alcyonium gracllimum*	Clone	Yang et al., 2013
		Zoanthid *Palythoa australiae*	Clone	Sun et al., 2014
*Micromonosporaceae*	*Micromonospora*	*Fungia scutaria*	Clone	Lampert et al., 2008
		*Platygyra lamellina*	Clone	Lampert et al., 2008
		*Antipathes dichotoma*	Isolate	Zhang et al., 2012
		*Tubastraea coccinea*	Clone	Yang et al., 2013
		*Acropora millepora*	Isolate	Li et al., 2014b
		*Galaxea fascicularis*	Isolate	Li et al., 2014b
		*Porites lutea*	Isolate	Li et al., 2014b
		*Scleronephthya* sp.	Isolate	Sun et al., 2012
		*Porites lutea*	Clone	This study
	*Verrucosispora*	gorgonian corals	Isolate	Zhang et al., 2013
	*Salinispora*	*Nephthea* sp.	Isolate	Ma et al., 2013
*Acidimicrobiales_Incertae_Sedis*	*Candidatus*_Microthrix	*Alcyonium gracllimum*	Clone	Yang et al., 2013
		*Tubastraea coccinea*	Clone	Yang et al., 2013
		*Porites lutea*	Clone	This study
*Corynebacteriaceae*	*Corynebacterium*	*Fungia granulose*	Isolate	Ben-Dov et al., 2009
		*Alcyonium gracllimum*	Clone	Yang et al., 2013
		*Tubastraea coccinea*	Clone	Yang et al., 2013
		Zoanthid *Palythoa australiae*	Clone	Sun et al., 2014
		*Porites lutea*	Clone	This study
***Cryptosporangiaceae***	***Fodinicola***	*Porites lutea*	Clone	This study
*Dermabacteraceae*	*Brachybacterium*	*Acropora digitifera*	Isolate	Nithyanand and Pandian, 2009
		*Galaxea fascicularis*	Isolate	Li et al., 2014b
		*Porites lutea*	Isolate	Li et al., 2014b
		*Porites lutea*	Clone	This study
*Microbacteriaceae*	*Agrococcus*	gorgonian corals	Isolate	Zhang et al., 2013
		*Porites lutea*	Clone	This study
	***Candidatus*****_Aquiluna**	*Porites lutea*	Clone	This study
	*Curtobacterium*	*Acropora digitifera*	Isolate	Nithyanand et al., 2011b
	*Leucobacter*	*Siderastrea sidereal*	Isolate	Cardenas et al., 2012
	*Microbacterium*	*Siderastrea sidereal*	Isolate	Cardenas et al., 2012
		*Tubastraea coccinea*	Clone	Yang et al., 2013
		*Porites lutea*	Isolate	Chen et al., 2014
		*Acropora millepora*	Isolate	Li et al., 2014b
		*Galaxea fascicularis*	Isolate	Li et al., 2014b
		*Porites lutea*	Clone	This study
	***Yonghaparkia***	*Porites lutea*	Clone	This study
*Micrococcaceae*	*Arthrobacter*	Stony coral	Isolate	Shnit-Orland and Kushmaro, 2009
		*Porites lutea*	Clone	This study
	*Kocuria*	*Acropora digitifera*	Isolate	Nithyanand et al., 2011b
		*Porites lutea*	Isolate	Chen et al., 2014
		Zoanthid *Palythoa Australia*	Clone	Sun et al., 2014
		*Porites lutea*	Clone	This study
	*Micrococcus*	*Acropora digitifera*	Isolate	Nithyanand et al., 2011b
		*Galaxea fascicularis*	Isolate	Li et al., 2014b
		*Porites lutea*	Clone	This study
	*Rothia*	*Platygyra carnosus*	Isolate	Chiu et al., 2012
		*Porites lutea*	Clone	This study
	***Tersicoccus***	*Porites lutea*	Clone	This study
*Gordoniaceae*	*Gordonia*	*Scleronephthya* sp.	Isolate	Sun et al., 2012
		*Alcyonium gracllimum*	Clone	Yang et al., 2013
		*Tubastraea coccinea*	Clone	Yang et al., 2013
		*Galaxea fascicularis*	Isolate	Li et al., 2014b
		*Acropora millepora*	Isolate	Li et al., 2014b
		*Porites lutea*	Isolate	Li et al., 2014b
*Jiangellaceae*	*Jiangella*	*Acropora millepora*	Isolate	Li et al., 2014b
		*Galaxea fascicularis*	Isolate	Li et al., 2014b
*Promicromonosporaceae*	*Cellulosimicrobium*	*Acropora millepora*	Isolate	Li et al., 2014b
		*Porites lutea*	Isolate	Li et al., 2014b
	*Myceligenerans*	*Fam. Caryophillidae*	Isolate	Sarmiento-Vizcaíno et al., 2015
*Tsukamurellaceae*	*Tsukamurella*	*Galaxea fascicularis*	Isolate	Li et al., 2014b

*The genera firstly reported in this study were shown in bold*.

According to our summary (Table 4), genera *Agrococcus, Amycolatopsis, Arthrobacter, Brachybacterium, Brevibacterium, Candidatus*_Microthrix, *Corynebacterium, Cellulosimicrobium, Cellulomonas, Dermatophilus, Dietzia, Gordonia, Janibacter, Jiangella, Kocuria, Kytococcus, Microbacterium, Micromonospora, Micrococcus, Mycobacterium, Nocardioides, Nocardiopsis, Propionibacterium, Pseudonocardia, Rhodococcus, Rothia, and Streptomyces* were detected in diverse coral species including scleractinian corals, such as *Acropora digitifera* (Nithyanand and Pandian, 2009; Nithyanand et al., 2011b), *P. lutea* (Li et al., 2014b; Sun et al., 2014) and *Galaxea fascicularis* (Li et al., 2014b), and gorgonian corals, *Siderastrea sidereal* (Cardenas et al., 2012) and *Platygyra carnosus* (Chiu et al., 2012). Most of them were present also in other marine organisms, such as sponges (Kim and Fuerst, 2006; Zhang et al., 2006; Selvin et al., 2009; Abdelmohsen et al., 2010, 2014; Schneemann et al., 2010; Sun et al., 2010; Webster and Taylor, 2012; Vicente et al., 2013), mollusks (Romanenko et al., 2008; Peraud et al., 2009), fishes (Sheeja et al., 2011), seaweeds (Lee, 2008; Singh and Reddy, 2013), seagrasses (Ravikumar et al., 2012), and sea cucumber (Kurahashi et al., 2009). Moreover, some of these widely distributed groups were considered as the bioactive compounds producers (Fiedler et al., 2005; Tabares et al., 2011; Margassery et al., 2012; Vicente et al., 2013; Manivasagan et al., 2014; Valliappan et al., 2014; EIAhwany et al., 2015), and probably take part in nitrogen (Su et al., 2013) and phosphorus (Sabarathnam et al., 2010) biogeochemical cycles. Whether they play these functional roles in corals *in situ* need to be further investigated.

### Comparison of actinobacterial communities in the corals and in the ambient seawater

Comparing the actinobacteria communities between in *P. lutea* and in the surrounding seawater will help us to understand the source of coral associated actinobacteria, and the interaction between the bacteria in sea water and in corals. Consisted with previous study on bacteria communities (Li et al., 2014a), the *P. lutea* associated actinobacteria communities were significantly different from which in the ambient seawater (Figure 3). Groups such as *Propionibacteriaceae, Micromonosporaceae*, and *Micrococcaceae*, were specifically associated with the corals rather than in the ambient seawater, where they originated from should be in doubt. Whether the wide distributed groups such as Sva0996_marine_group, OM1_clade, *Microbacteriaceae* and *Ilumatobacter* travel between the ambient seawater and the corals need to be investigated.

When researchers make a general observation of the whole bacterial communities, which were observed significantly different in coral mucus, tissue, and skeleton (Rohwer et al., 2002; Bourne and Munn, 2005; Sweet et al., 2011; Lee et al., 2012). However, it is unclear whether actinobacteria has a similar distribution pattern. In this study, both the hierarchical cluster analysis (Figure 3) and the RDA analysis (Figure 4) showed that the actinobacteria communities from the same compartment tended to cluster together. The distinct physiochemical microenvironments provided by corals probably is one of the causes (Le Tissier, 1990; Brown and Bythell, 2005; Sweet et al., 2011; Tremblay et al., 2011). Only a small fraction of OTUs (22 out of 299 OTUs in the coral libraries) was common in the coral mucus, tissue, and skeleton libraries in this study (Table 2). This result suggested that these members might have capabilities to adapt to different micro-environments in divergent compartments of *P. lutea*. A large amount of the OTUs was specifically associated with a certain coral compartment. Whether and how the properties of distinct actinobacteria assemblages in different coral compartments actually contribute to the close relationship constructed between these associates and corals should be addressed from a functional perspective.

### Relationship of environmental factors and the *P. lutea*-associated actinobacteria

It is different from previous conclusion of the distribution of coral-associated bacteria (Chen et al., 2011; Li et al., 2014a), actinobacteria associated with *P. lutea* did not show the apparent seasonal dynamic variations. We suggest that the actinobacteria compositions are relatively stable in distinct compartments in *P. lutea*. In addition, none of the environmental factors analyzed in this study was determined as the most significant influence on the actinobacteria communities. Even so, some genera were found closely correlated with specific environmental factors. For instance, *Propionibacterium* showed negatively correlation with dissolved oxygen, probably due to its capability of living in the anaerobic conditions (Patrick and McDowell, 2012). Moreover, the OTUs0003 and 0004 affiliated with *Propionibacteriaceae* was present in almost all 12 clone libraries with a very high abundance. Whether they are true symbionts, and what functions they play are worth further research.

## Conclusion

The diversity and distribution of coral-associated actinobacteria were first comprehensively investigated in this study. Highly diverse actinobacteria was revealed in the 16S rRNA gene clone libraries of scleractinian coral *P. lutea* in the South China Sea. Twelve *Actinobacteria* genera were detected in corals for the first time as well as a large number of unclassified groups. The actinobacterial community compositions were distinct in *P*. *lutea* and in the surrounding seawater. Furthermore, the higher similarity of actinobacteria composition was observed in the same compartment (i.e., mucus, tissue, or skeleton) of *P*. *lutea*. This study will help attracting the attentions on the ecological role of actinobacteria in corals besides the natural products bioprospecting.

### Conflict of interest statement

The authors declare that the research was conducted in the absence of any commercial or financial relationships that could be construed as a potential conflict of interest.
