# Supplementary material for: Diversity and distribution of Actinobacteria associated with reef coral Porites lutea
Source: Front Microbiol. 2015 Oct 21;6:1094. doi: 10.3389/fmicb.2015.01094 (PMC4612714; doi:10.3389/fmicb.2015.01094)
Supplement: Supplementary file 1 [file DataSheet1.DOC]

**Diversity and distribution of *Actinobacteria* associated with reef coral *Porites lutea***

**Weiqi Kuang^1,2†^, Jie Li^1†^, Si Zhang^1^, Lijuan Long^1^**

^1^ CAS Key Laboratory of Tropical Marine Bio-resources and Ecology, RNAM Center for Marine Microbiology, South China Sea Institute of Oceanology, Chinese Academy of Sciences, Guangzhou, Guangdong, P. R. China

^2^ University of Chinese Academy of Sciences, Beijing, P. R. China

**^†^ These authors contribute equally to this work.**

**Corresponding author:** Lijuan Long, CAS Key Laboratory of Tropical Marine Bio-resources and Ecology, RNAM Center for Marine Microbiology, South China Sea Institute of Oceanology, Chinese Academy of Sciences, Xingangxi Road 164, Guangzhou, Guangdong, P. R. China.

longlj@scsio.ac.cn

**Keyword: diversity, actinobacteria, 16S rRNA gene, *Porites lutea*, temporal and spatial distribution**

**Running title**: *Porites lutea*-associated actinobacteria

**Table S1**￨Summary of the redundancy analysis. Automatic forward selection was performed with Monta Carlo permutation tests.

| Axes | 1 | 2 | 3 | 4 |
| --- | --- | --- | --- | --- |
| Eigenvalues | 0.248 | 0.085 | 0.042 | 0.015 |
| Species-environment correlations | 0.845 | 0.651 | 0.587 | 0.604 |
| Cumulative percentage variance | | | | |
| of species data | 24.8 | 33.3 | 37.4 | 38.9 |
| of species-environment relation | 63.7 | 85.5 | 96.2 | 100.0 |
| Sum of all canonical eigenvalues | 0.389 | | | |

**Figure S1**￨Venn diagram showing the shared OTUs (3% distance level)

**(A)￨**The OTUs shared by different coral compartments and by the surrounding seawater.

**(B)￨**The OTUs shared by the coral samples collected in different months.


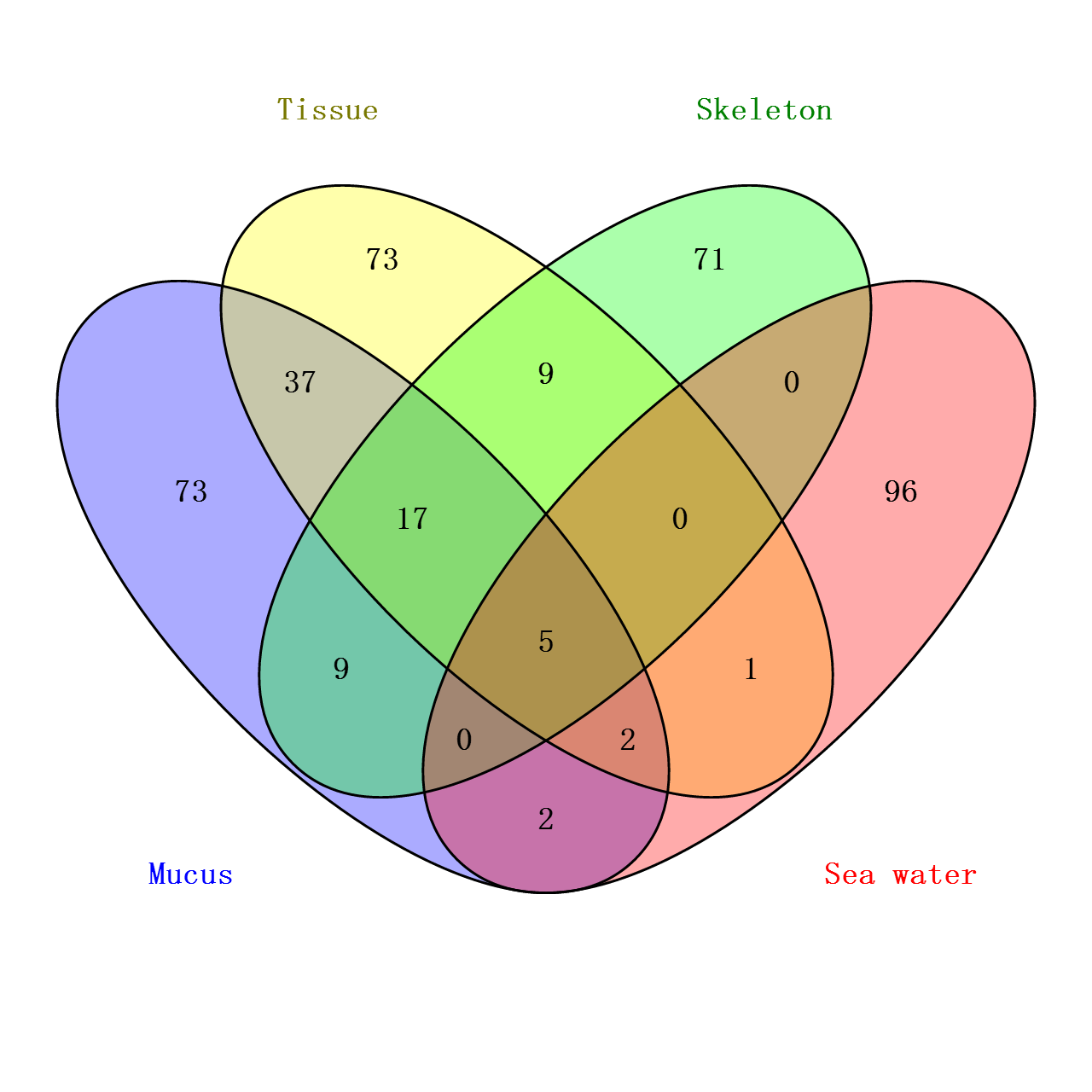

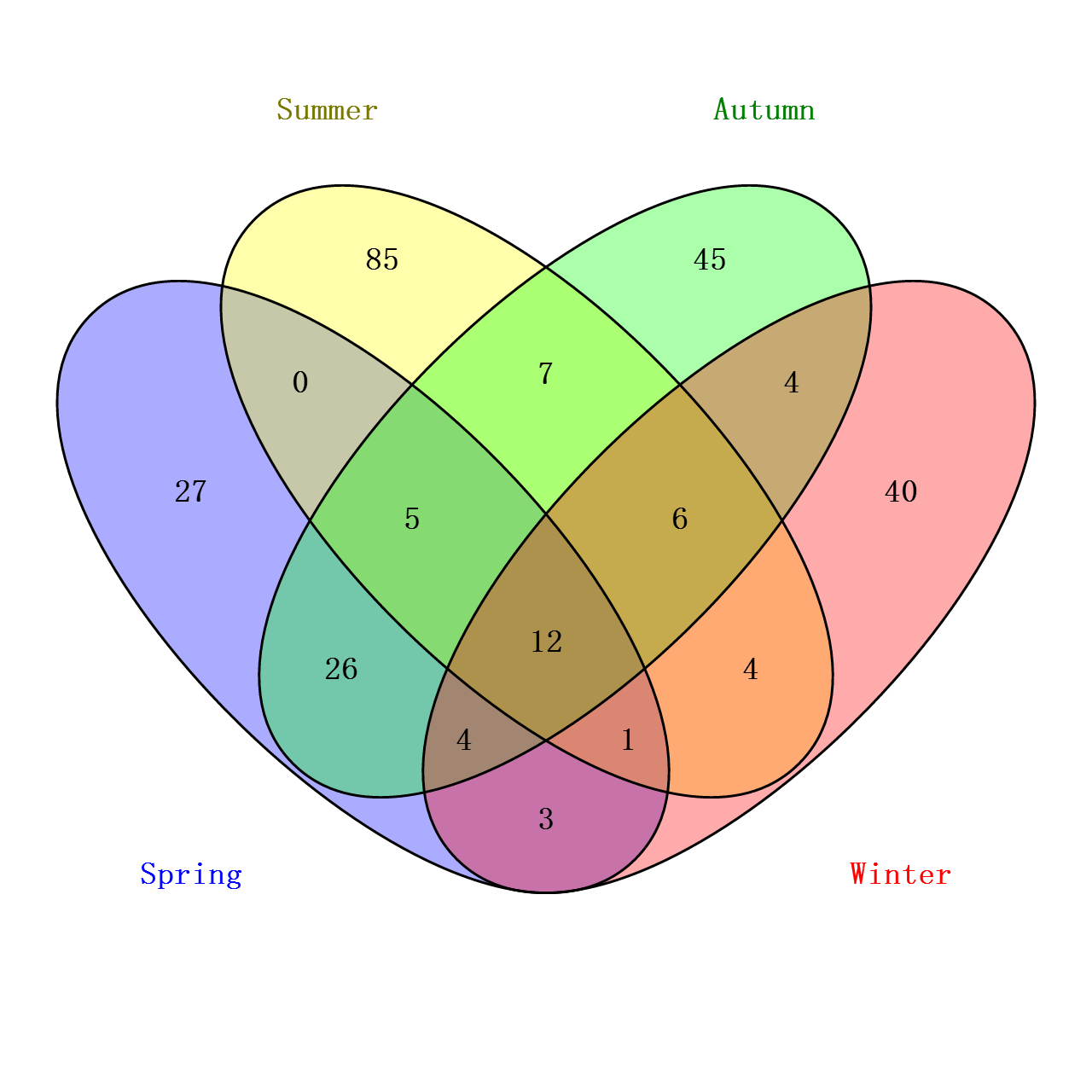


**A B**

**Data S1**

The GenBank accession numbers of actinobacteria 16S rRNA gene sequences were as follows:

KP303715–KP303719; KP303722–KP303736; KP303738–KP303741; KP303743–KP303746; KP303748; KP303749; KP303751; KP303752; KP303754–KP303759; KP303761–KP303764; KP303766–KP303770; KP303772; KP303773; KP303775–KP303790; KP303793; KP303796–KP303798; KP303800–KP303813; KP303815–KP303822; KP303824–KP303831; KP303833–KP303838; KP303840–KP303860; KP303862–KP303868; KP303870; KP303872–KP303881; KP303883; KP303887–KP303892；KP303894–KP303897; KP303899–KP303901; KP303903–KP303924; KP303926; KP303928–KP303940; KP303942–KP303956; KP303958; KP303961–KP303964; KP303966; KP303967; KP303969–KP303973; KP303975–KP303978; KP303980–KP303985; KP303987–KP303991; KP303994; KP303996–KP304003; KP304005; KP304008; KP304010–KP304016; KP304018–KP304023; KP304025–KP304050; KP304052–KP304063; KP304065; KP304066; KP304068–KP304072; KP304075; KP304077–KP304080; KP304082–KP304092; KP304094–KP304112; KP304114–KP304120; KP304122–KP304128; KP304130–KP304135; KP304137–KP304140; KP304143–KP304159; KP304161; KP304163; KP304165–KP304169; KP304171–KP304181; KP304183–KP304187; KP304190–KP304200; KP304202–KP304206; KP304208–KP304210; KP304213–KP304216; KP304218–KP304220; KP304222–KP304227–KP304230; KP304232–KP304235; KP304237; KP304239–KP304241; KP304243–KP304244; KP304246–KP304251; KP304253–KP304266; KP304269–KP304270; KP304273; KP304274; KP304276–KP304279; KP304282–KP304305; KP304307–KP304309; KP304311; KP304312; KP304314–KP304317; KP304319; KP304320; KP304322–KP304326; KP304328–KP304330; KP304333–KP304335; KP304337; KP304338; KP304341–KP304350; KP304352; KP304354–KP304358–KP304361; KP304364; KP304365; KP304367; KP304369–KP304378; KP304382; KP304383; KP304385; KP304386; KP304388; KP304389; KP304392; KP304396–KP304404; KP304407; KP304409; KP304411–KP304413; KP304420–KP304422; KP304426–KP304429; KP304431; KP304433; KP304439–KP304442; KP304445; KP304448; KP304450; KP304453–KP304455; KP304457; KP304459; KP304461; KP304463; KP304466–KP304468; KP304471; KP304472; KP304479; KP304483; KP304487; KP304488; KP304492; KP304493; KP304495; KP304498–KP304503; KP304505; KP304508; KP304510; KP304511; KP304517; KP304519–KP304521; KP304525–KP304528; KP304531–KP304536; KP304538; KP304540; KP304541; KP304543; KP304547; KP304549; KP304551–KP304560; KP304562; KP304565–KP304568; KP304571–KP304575; KP304577–KP304580; KP304582–KP304592; KP304594–KP304596; KP304601–KP304605; KP304607; KP304608; KP304611–KP304616; KP304618–KP304625; KP304628–KP304630; KP304634; KP304637–KP304640; KP304642; KP304643; KP304645; KP304646; KP304649–KP304655; KP304658; KP304659; KP304661–KP304663; KP304665; KP304668–KP304672; KP304676; KP304680; KP304681; KP304683; KP304685–KP304686; KP304688; KP304691; KP304692; KP304694–KP304696; KP304699–KP304701; KP304704; KP304705; KP304707–KP304709; KP304714–KP304728; KP304730; KP304732; KP304736–KP304743; KP304745–KP304752; KP304754–KP304774; KP304776; KP304778–KP304790; KP304792–KP304805; KP304807; KP304810–KP304813; KP304815; KP304816; KP304818–KP304821; KP304823–KP304826; KP304828–KP304833; KP304835–KP304839; KP304842; KP304844–KP304851; KP304853; KP304856; KP304858–KP304869; KP304871–KP304896–KP304898; KP304901–KP304905; KP304907; KP304909; KP304912; KP304914; KP304915; KP304917–KP304935; KP304937; KP304939–KP304944; KP304946–KP304954; KP304956; KP304958–KP304960; KP304962–KP304965; KP304968–KP304971; KP304973–KP304979; KP304982–KP304985; KP304987; KP304988; KP304990; KP304994; KP304996–KP304998; KP305000–KP305002; KP305004; KP305006; KP305008–KP305010; KP305014; KP305017–KP305022; KP305024; KP305025; KP305027–KP305030; KP305032–KP305037; KP305040; KP305041; KP305043–KP305048; KP305051–KP305055; KP305057–KP305059; KP305061–KP305064; KP305066–KP305073; KP305075; KP305077–KP305086; KP305088; KP305091; KP305094–KP305099; KP305102 – KP305104; KP305106 – KP305108; KP305111–KP305119; KP305122; KP305123; KP305125–KP305134; KP305137; KP305138; KP305141–KP305143; KP305145–KP305150; KP305152–KP305157; KP305159–KP305162; KP305164–KP305166; KP305168; KP305169; KP305171–KP305174; KP305176; KP305177; KP305179–KP305181; KP305183–KP305188; KP305190–KP305198; KP305200–KP305202; KP305204–KP305217; KP305219–KP305223; KP305226–KP305228; KP305230–KP305234; KP305237; KP305239–KP305241; KP305244–KP305252; KP305254–KP305256; KP305259–KP305261; KP305263; KP305264; KP305266; KP305267; KP305270; KP305272–KP305279; KP305281–KP305289; KP305290–KP305301; KP305303–KP305308; KP305311–KP305319; KP305321–KP305323; KP305325; KP305326; KP305328–KP305332; KP305334; KP305345; KP305347–KP305350; KP305352–KP305358; KP305360; KP305362–KP305367; KP305369–KP305372; KP305374–KP305377; KP305382; KP305383; KP305385–KP305387; KP305389; KP305391–KP305397; KP305399–KP305401; KP305405; KP305407–KP305409; KP305411–KP305412; KP305414–KP305422; KP305424–KP305431; KP305433; KP305437–KP305438; KP305440–KP305448; KP305450–KP305455; KP305457; KP305458; KP305462–KP305464; KP305466–KP305470; KP305472–KP305479; KP305481; KP305484; KP305486; KP305488; KP305490–KP305492; KP305494; KP305496; KP305497; KP305499; KP305500; KP305502–KP305505; KP305507; KP305509; KP305511; KP305513–KP305515; KP305517; KP305519; KP305521–KP305526; KP305528–KP305533; KP305535; KP305536; KP305538; KP305541–KP305549; KP305552–KP305555; KP305557–KP305566; KP305568; KP305571; KP305573; KP305576–KP305583; KP305585; KP305586; KP305589–KP305592; KP305595–KP305597; KP305600; KP305602–KP305606; KP305608; KP305615; KP305616; KP305619–KP305620; KP305622–KP305624; KP305628; KP305631; KP305632; KP305634; KP305638; KP305640; KP305641; KP305643; KP305647; KP305650; KP305652–KP305656; KP305658; KP305660–KP305689; KP305691–KP305707; KP305710–KP305720; KP305722–KP305724; KP305726–KP305744; KP305746–KP305754; KP305756–KP305759; KP305761–KP305781; KP305783–KP305785; KP305787; KP305789–KP305800; KP305802–KP305807; KP305809–KP305822; KP305824–KP305828; KP305830–KP305842; KT714248–KT715013
